# Association between tumour necrosis factor-a polymorphism and cervical cancer in Lagos State, Nigeria

**DOI:** 10.3332/ecancer.2025.1845

**Published:** 2025-02-12

**Authors:** Sarah O John-Olabode, Ifeoma C Udenze, Adebola A Adejimi, Obiefuna Ajie, Kehinde S Okunade

**Affiliations:** 1 Department of Haematology and Blood Transfusion, College of Medicine, University of Lagos, PMB 12003, Lagos, Nigeria; 2 Department of Clinical Pathology, College of Medicine, University of Lagos, PMB 12003, Lagos, Nigeria; 3 Department of Community Health, College of Medicine, University of Lagos, PMB 12003, Lagos, Nigeria; 4 Department of Obstetrics and Gynaecology, College of Medicine, University of Lagos, PMB 12003, Lagos, Nigeria

**Keywords:** cervical cancer, Nigeria, screening, polymorphism, TNF-α

## Abstract

**Background:**

The data on tumour necrosis factor-α (TNF-α) promoter gene polymorphism in the African population are relatively limited, especially in Nigerian women.

**Objectives:**

This study aimed to determine the prevalence and allele distribution of three TNF-α promoter gene SNPs loci – rs361525 (−238 G>A), rs1799964(−1031 T>C) and rs1800629 (−308 G>A) in women with cervical cancer (CC) and then evaluated the association between TNF-α SNPs and CC among women in Lagos, Nigeria.

**Methods:**

This is a cross-sectional study of 75 unmatched human immunodeficiency virus (HIV)-infected and uninfected women with and without CC enrolled from October 2021 to January 2023 at the gynaecological oncology, cytology, adult HIV and blood donor clinics of the Lagos University Teaching Hospital. About 5 mL of peripheral blood was collected from each participant for total Deoxyribonucleic acid extraction, primer synthesis and genotyping. The probability of developing CC based on the given SNP genotype was expressed as an odds ratio (OR) with a 95% confidence interval. Allelic frequency deviations from Hardy–Weinberg equilibrium were calculated using chi-square, and the statistical significance level was considered as two-tailed and set at *p* ≤ 0.05.

**Results:**

Our study found that TNF-α −1031 T>C polymorphism was significantly associated with increased CC risk in HIV-negative women (HIV+/CC-; OR = 1.4, 95%CI 0.23–8.42, *p* = 0.03 and HIV-/CC-; OR = 1.37, 95%CI 0.01–1.68, *p* = 0.03) while the −308A>G A allele was also significantly associated with CC in HIV-positive women (OR = 1.33, 95%CI = 0.23–7.75).

**Conclusion:**

We observed that HIV-negative and HIV-positive women who carry the C allele of −1031T>C and the A allele of −308G>A *TNF-a* promoter gene loci, respectively, are more susceptible to CC. We were also able to show protective linkages for the minor allele of the three SNPs of interest suggesting the potential of TNF-a as a surrogate marker for CC screening in addition to human papillomavirus primary testing. Further studies are required to determine the association between host factors and TNF-a polymorphism to harness the diagnostic and therapeutic advantage these associations will provide in the management of CC.

## Introduction

Cervical cancer (CC) is the most common gynaecological cancer with over half a million new cases and over a quarter of a million deaths reported globally in 2020 [1]. Despite significant advances in the diagnosis, treatment and prevention of CC, the attributable burden of disease due to CC remains high especially in low- and middle-income countries [2]. Indeed, recent data shows the highest number of new cases and mortality due to CC is in the sub-Saharan Africa region [1, 3].

The pathogenesis of CC has been closely linked with various risk factors, including environmental, lifestyle and host factors. Women living with human immunodeficiency virus (HIV) infection are more susceptible to CC [4, 5]. According to the World Health Organisation, HIV-positive women have a six-fold higher risk of developing CC compared to women without HIV, with approximately 5% of all CC cases attributed to HIV [3]. CC is regarded as one of the acquired immunodeficiency syndromes-defining cancers [6]. HIV causes immunosuppression which increases the risk of human papillomavirus (HPV) infection [7]. Women living with HIV are more likely to have HPV co-infection and this creates a vicious cycle with HIV-promoting HPV-induced cervical carcinogenesis [3]. The interaction between HIV, HPV and host factors has been recognised to be involved in the process of cervical carcinogenesis [7]. In association with the synergy between HIV and HPV, inherited genetic predisposition in the host also appears to play a significant role in the development of invasive CC with accumulating evidence suggesting that polymorphisms in key regulatory genes (including tumour suppressor and other immune-related genes) play an important role in the susceptibility to HPV infection [8–15].

Tumour necrosis factor-α (TNF-α) gene single nucleotide polymorphisms (SNPs) have been associated with increased susceptibility to CC and these polymorphisms appear to be concentrated in the gene promoter region [16, 17]. Located on human chromosome 6 (6p21.3) in the Human Leukocyte Antigen (HLA) region between the Class I HLA-B and the Class II HLA-DR loci, the *TNF-α gene* codes for the TNF-α protein [16, 17]. TNF-α is a pro-inflammatory cytokine implicated in the clearance of HPV *in vivo* through the downregulation of viral gene transcription and promoting the host’s inflammatory response to eradicate the HPV. Additionally, TNF-α promotes apoptosis in CC cells [18–20]. While TNF-α is recognised to be of some benefit in CC through its proapoptotic role, some evidence has linked SNPs in the *TNF-α promoter genes* with CC and other solid tumours such as breast cancer in various racial populations [21, 22]. While some studies have shown some association between *TNF-α promoter gene* polymorphism and CC, this remains controversial as other studies observed no such relationship [20–27]. SNPs rs361525 (−238 G>A), rs1799964 (−1031 T>C) and rs1800629 (−308 G>A) have been studied extensively and notably rs1800629 (−308) polymorphism has been associated with the development of other solid tumours such as breast cancer [24–28].

Taken together, studying the genetic variants in CC is of diagnostic and prognostic value. Identifying genetic variants will help in understanding the biology of CC and serve as potential biomarkers and therapeutic targets. Although, CC screening using cytology has improved early disease detection, including genetic screening will provide information for disease characterisation, especially in high-risk women and improve disease monitoring and cost-effectiveness of CC management. The data on TNF-α promoter gene polymorphism in the African population are relatively limited, especially in Nigerian women. This study aimed to determine the prevalence and allele distribution of three TNF-α promoter gene SNPs loci – rs361525 (−238 G>A), rs1799964 (−1031 T>C) and rs1800629 (−308 G>A) in women with CC and then evaluated the association between TNFα SNPs and CC among women in Lagos, Nigeria.

## Materials and methods

### Study design and setting

This is a cross-sectional study conducted between October 2021 and January 2023 at the gynaecological oncology, cytology, adult HIV and blood donor clinics of the Lagos University Teaching Hospital (LUTH). LUTH is the leading tertiary healthcare facility in Lagos, Southwest Nigeria. It primarily serves as a specialised referral center for both public and private hospitals in Lagos and the neighbouring Ogun and Oyo States.

### Study population

Eligible participants were 100 unmatched HIV-infected and HIV-negative women with and without CC enrolled at the study clinics. Inclusion criteria were HIV-positive women with pathologically confirmed cervical squamous cell carcinoma enrolled from the gynaecological oncology and cytology clinics (case group) and otherwise healthy HIV-positive and HIV-negative women randomly selected from the HIV and donor clinics during the same study period (comparison group). Exclusion criteria included history of other malignancies, refusal of consent at enrolment or withdrawal of consent during the study,

### Study procedure

**Figure 1.**
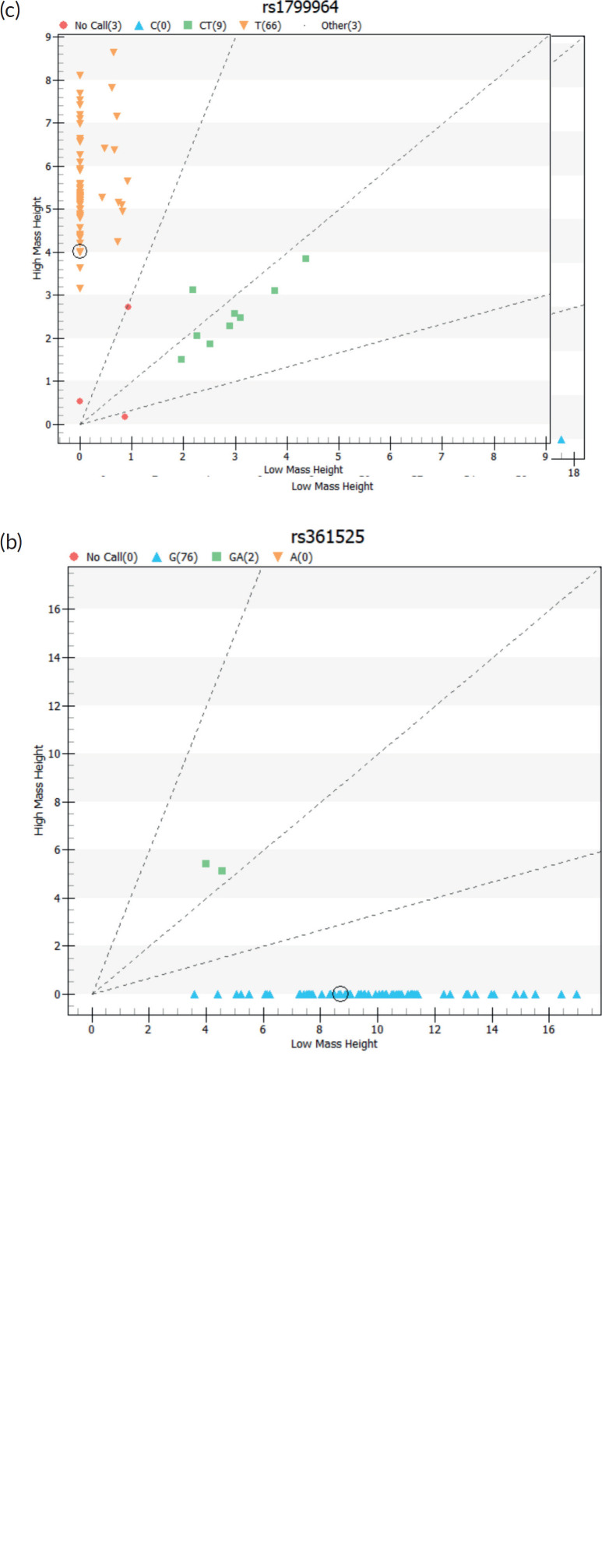
Cell cluster plots of TNF-α SNPs genotyping.

After ascertaining eligibility and collecting relevant sociodemographic and clinical information from each participant, about 4–5 mLs of peripheral blood samples were collected into ethylene diamine tetra acetic acid bottles at enrolment for Deoxyribonucleic acid (DNA) analysis. Total DNA was extracted from the individual’s buffy coats using the Quick-DNA ™ MiniPrep kit from Zymo Research (ZymoResearchCorp. Irvine, CA, USA, Catalog number: D3024) according to the manufacturer’s instructions and then stored at −20°c. Total DNA quality was assessed using NanoDrop 2000 (Thermo Fisher Scientific Inc., Waltham, MA, USA) using a 1.7–2.0 optical density range for the genotyping. The final sample concentration was diluted to 50 ng/μL and sent to Inqaba Biotechnical Industries (Pty) Ltd. (Pretoria, South Africa) for primer synthesis and genotyping using the Agena MassArray system (Agena Bioscience San Diego, CA, USA) (Figure 1, Table 1).

### Statistical analysis

Statistical analysis was performed using SPSS version 29.0 (IBM Corp., Armonk, NY, USA). The Shapiro-Wilk method was used to test quantitative variable normality. Continuous variables were expressed as mean ± standard deviation (SD), and categorical variables were expressed in percentages and frequencies. Significant differences between the group continuous variables were tested with the Student’s *T*-test and analysis of variance while Pearson’s chi-square and Fisher’s exact test were used to evaluate differences in genotype distribution between subgroups. The probability of developing CC based on the given SNP genotype was expressed as an odds ratio (OR) with a 95% confidence interval (95%CI) [29]. Allelic frequency deviations from Hardy–Weinberg equilibrium (HWE) were calculated using chi-square [30]. The statistical significance level was considered as two-tailed and set at *p* ≤ 0.05.

## Results

**Figure 2:**
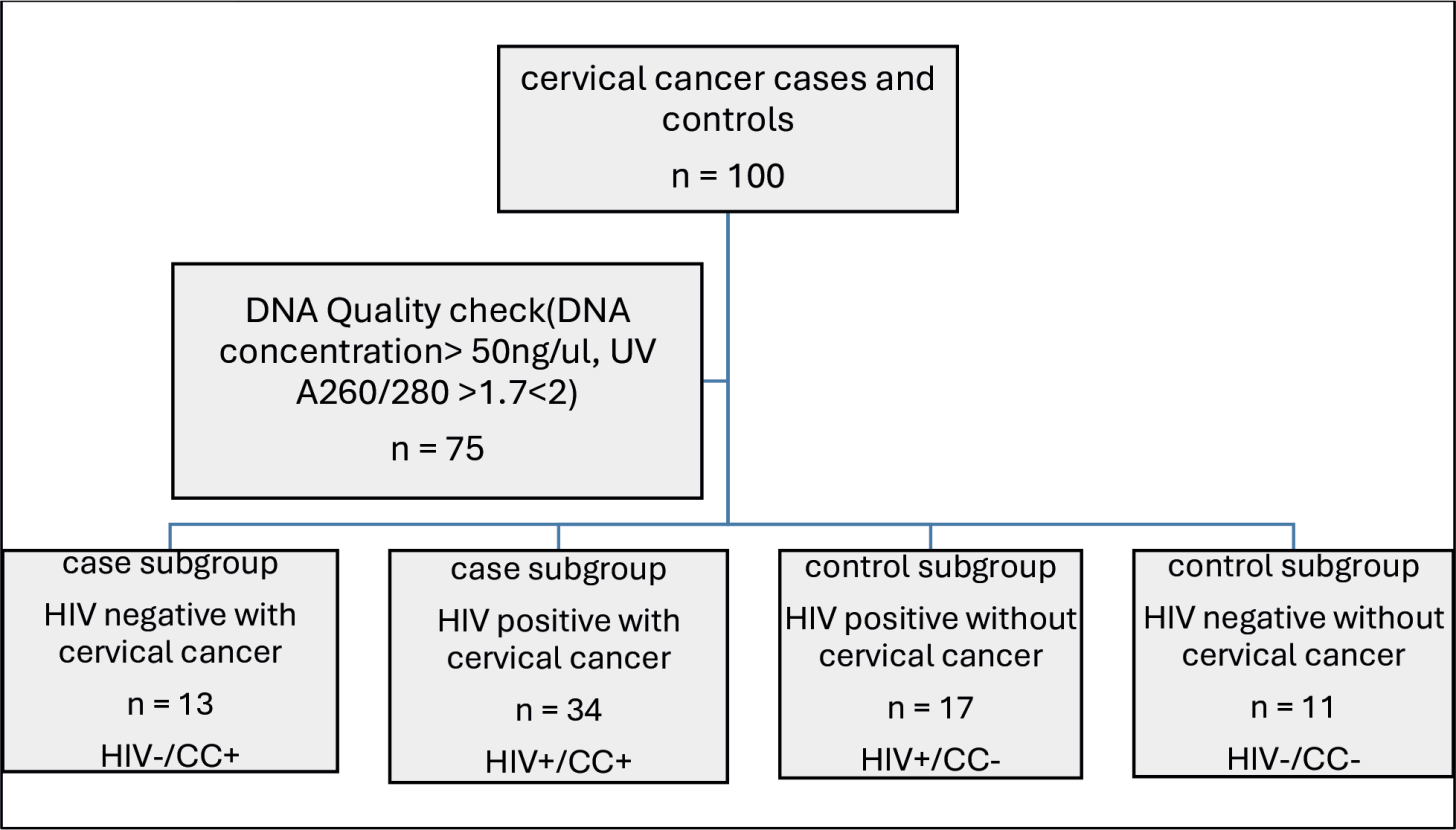
Flowchart of Participants’ stratification

Out of the 100 participants recruited for this study, only 75 participants' DNA samples met the quality criteria for genotyping. The participants were further stratified into subgroups, namely HIV negative with CC (HIV–/CC+; *n* = 13, 17.1%), HIV positive with CC (HIV+/CC+; *n* = 34, 44.7%), HIV positive without CC (HIV+/CC–; *n* = 17, 22.4%) and HIV negative without CC (HIV–/CC–; *n* = 11, 14.5%) (Figure 2).

Overall, the ages of participants with CC were significantly higher than that of their comparison groups (*p* < 0.01) but no significant differences were observed in patients ages at menarche and coitarche between the subgroups – HIV–/CC+ versus HIV+/CC+ (57.62 ± 13.35 versus 58 ± 9.67 years, *p* = 0.94) and HIV+/CC– versus HIV–/CC– (43.18 ± 9.31 versus 41.82 ± 7.74 years, *p* = 0.68). The key demographic data are summarised in Table 2.

### Genotypes and allelic distribution of the studied TNFα SNPs

The allele distribution and genotype frequencies of −308/−238/−857 loci of *TNF-α* promoter among CC (with/without HIV) and the comparison groups are shown in Table 2. For *TNF-α* -308G>A, TNF-α −238G>A and *TNF-α* – 1031T>C SNPs, the genotype distributions were consistent with HWE in both the CC and comparison groups (Table 3).

### TNF-α SNP genotyping test results by group

There was no significant difference in the *TNF-α* −308 homozygous and heterozygous genotype (GG/GA) between the CC cases and the comparators (*p*^a^ = 0.63, *p*^b^ = 0.73 and *p*^c^ = 0.82, respectively, Table 3). We observed a higher distribution of the heterozygous GA genotype among the study group compared to the comparison group with 17% (8/47) in cases and 14.3% (4/28) in the comparators. Notably, the rare AA genotype was observed in the HIV+CC+ subgroup. The same was true in both the case and control groups for *TNF-α* -238(GG/GA) (*p*^a^ = 1.00, *p*^b^ = 0.33, respectively, and −1031(TT/CT) (*p*^a^ =1.00, *p*^b^ = 0.10 and *p*^c^ = 0.14, respectively) (Table 3).

### Allele frequency of TNF-α −308G/A, −238G/A and −857C/T in CC and comparison groups

Regarding allele distribution of −308G>A, the A allele frequencies of −308G>A in the CC group HIV-/CC+ and HIV+/CC- in the comparison group were 12% and 6%, respectively, (OR = 0.44, 95%CI = 0.06–3.16, *p* = 0.06). The frequency of the A allele in the CC group HIV+/CC+ (10%) was significantly higher than the HIV+/CC- in the comparison group (6%) (*p* = 0.04). The estimated OR was 1.33 (95%CI = 0.23–7.75), which indicated an increased risk for the development of CC in HIV-positive women carrying the A allele of TNF α-308G>A. Comparing the HIV-/CC+ in the CC group and the HIV-/CC- in the comparison group (12% versus 9%), there was a significant difference in A allele frequency (OR = 0.81, 95%CI = 0.13–4.88, *p* = 0.03) but as observed previously there was no increased risk of CC in HIV negative women carrying the A allele of −308G>A (Table 3).

The A allele frequencies of −238G>A showed a significant difference between HIV-/CC+ in the CC group and HIV+/CC- in the comparison group (4% versus 3%), respectively (OR = 0.75, 95%CI = 0.04–13.2, *p* = 0.01). The OR<1 supports our observation of the A allele being a protective factor against CC in HIV-negative women. The HIV+/CC+ and HIV-/CC- comparison groups carried only the G allele (Table 3).

In Table 3, the C allele frequencies of −1031T>C of HIV-/CC+ in the CC group and comparison groups were significantly different (HIV+/CC-; OR = 1.4, 95%CI 0.23–8.42, *p* = 0.03 and HIV-/CC-; OR = 1.37, 95%CI 0.01–1.68, *p* = 0.03). The presence of the C allele had a significant association with CC in HIV-negative women (ORs = 1.4 and 1.37, respectively). In the HIV+CC+ and HIV+CC- subgroups, the C allele frequencies were 1% versus 9%, respectively, with OR<1 suggesting the presence of the C allele as a protective factor against CC in HIV-positive women (OR = 0.14, 95% CI 0.01–1.48 *p* = 0.08).

## Discussion

Our study evaluated the association between genotype and allele frequency at three SNP loci of *TNF-a* promoter rs361525 (−238 G>A), rs1799964(−1031 T>C) and rs1800629 (−308 G>A) and CC in Lagos State, Nigeria. We reported that the TNF-α −1031 T>C polymorphism was significantly associated with increased CC risk in HIV-negative women while the −308A>G A allele was also significantly associated with CC in HIV-positive women.

Contrary to previous studies [31, 32], our study revealed that the TNF-α −1031 T>C polymorphism was significantly associated with an increased risk of CC in HIV-negative women (OR = 1.4, 95%CI 0.23–8.42, *p* = 0.03). Our observations suggested that while HIV-negative women who carry the C allele in the *TNF-α* −1031 T>C gene locus are more susceptible to developing CC, the presence of the C allele offered protection from CC in HIV-positive women. Similar to evidence from the Asian population [21, 31, 33–35], in this study, the A allele of the −308G>A or −238G>A was found to be a protective factor for CC (OR = 0.72; 95% CI = 0.56–0.92 and OR = 0.75, 95%CI = 0.04–13.2) in HIV-negative women. Notably, the −308A>G A allele was found to be a risk factor for CC in HIV-positive women (OR = 1.33, 95%CI = 0.23–7.75). A review of previous literature reveals conflicting data on the association between −308G/A and −238G/A with CC in different or the same ethnic populations. While some studies conducted in Portuguese, Indian and Chinese women revealed an association between −308G>A and −238G>A and increased susceptibility to CC [16, 20, 36, 37], other studies carried out in women with diverse ethnicity including Mexican, Tunisian, American, South-African and Chinese populations reported the inheritance of the A allele of −308G>A or −238G>A and reduced the risk of developing CC [31, 33–35]. The possible explanations for the contradicting data in the different ethnic populations could be the diverse research methods used in these studies and most importantly the underlying host factors such as HIV infection. HIV infection causes a 6-fold rise in the risk of CC [3] due mostly to persistent HPV infection secondary to immune deficiency [38]. This has also been suggested by previous studies that reported that the host genetic background may facilitate HPV viral persistence in the uterine cervix as polymorphisms in coding regions of cytokine-like TNFA SNPs genes have been associated with susceptibility to some human diseases [14, 15]. However, we were not able to confirm this possible link in our present study as we did not assess the presence of genital high-risk HPV infection or its persistence in the enrolled participants.

TNF-α is one of the key inflammatory cytokines produced by macrophages that have been shown to play a crucial role in immune defense against pathogens including the control of HPV infection [21, 39]. TNF- α directly promotes the reduction of HPV gene transcripts, induces apoptosis in HPV infected/cancer cells and enhances inflammatory response to HPV. In addition to upregulating HPV presentation to effector T cells by antigen-presenting cells [40], evidence suggests* TNF- α* promoter region SNPs including rs361525 (−238 G>A), rs1799964(−1031 T>C) and rs1800629 (−308 G>A) may cause deregulation of TNF- α transcription resulting in varied TNF- α levels in circulation. A low TNF- α results in reduced HPV antigen presentation with persistence of HPV infection while high TNF-α levels result in enhanced inflammatory response that drives cervical carcinogenesis with increased susceptibility to CC [32]. Our hypotheses in the study were – first, the genotype frequency of *TNF-α* promoter region SNPs is higher in HIV-negative women with CC than in comparators. Second, the genotype frequency of *TNF-α* promoter region SNPs is higher in HIV-positive women with CC than in HIV-negative women with CC and controls. The results we observed showed that the frequency of *TNF-a* promoter SNPs rs361525 (−238 G>A), rs1799964 (−1031T>C) and rs1800629 (−308 G>A) (7.7% versus 5.9%, 23.1% versus 18.2% and 23.1 versus 18.2%, *p* = 0.82, 1 and 0.14, respectively) in CC subgroup were not significantly different enough to reject the null hypothesis. In addition, our data were suggestive of the minor A allele of −308G>A and 238G>A giving a protective advantage for CC in HIV-negative women while −1031T>C minor C allele increased susceptibility for CC in the same group.

The frequency distribution of rs361525 (−238 G>A), rs1799964(−1031 T>C) and rs1800629 (−308 G>A) alleles in the HIV-positive women with CC subgroup followed a similar pattern (0.0% versus 5.9%, 14.7% versus 11.8%, 2.9% versus 17.6%, *p* = 0.73, 0.33 and 0.10) with no statistically significant difference between the cases and comparators. In addition, our data were suggestive of the minor A allele of −1031T>C giving a protective advantage for CC in HIV-positive women while −308G>A minor A allele increased susceptibility for cervical in the same group. Our findings indicate the association of *TNF-a* promoter SNPs rs361525 (−238 G>A), rs1799964(−1031 T>C) and rs1800629 (−308 G>A) with increased susceptibility to CC is dependent on underlying host factor, most especially factors that impair the immune system. This could be a possible reason for the conflicting findings in previous studies that have investigated the link between *TNF-a* promoter gene polymorphism and the risk of CC. It is, therefore, important to interpret data in the context of underlying host factors.

The main strength of this study is that is the first research, to the best of our knowledge, that evaluated the association between genotype and allele frequency at three SNP loci of *TNF-a* promoter rs361525 (−238 G>A), rs1799964(−1031 T>C) and rs1800629 (−308 G>A) and the risk of CC in Lagos State, Nigeria. The study, however, had several limitations. First, there was an age mismatch between the cases and comparison group thus making it probable that the protective advantage of SNPs of interest may have been overestimated as potential cases are currently classified as comparators. Second, the small sample size used in the study could potentially reduce the power to detect associations of minor alleles with CC. Third, the use of cervicovaginal washings instead of whole blood could have provided more information on the molecular dynamics within the cancer cells. Fourth, we did not investigate HPV infection; therefore, we could not directly examine any association between *TNF-a* promoter gene polymorphism and HPV viral persistence. Finally, there was no measurement of TNF-a serum level which could have given us an insight into the expression and functionality of the three *TNF-a* SNPs of interest. Based on these observations, we suggest that future research with a more robust sample size should be designed to validate the association between the identified *TNF-a* promoter gene polymorphisms and susceptibility to CC.

## Conclusion

We observed that HIV-negative and HIV-positive women who carry the C allele of −1031T>C and the A allele of −308G>A *TNF-a* promoter gene loci, respectively, are more susceptible to CC. We were also able to show protective linkages for the minor allele of the three SNPs of interest and this would suggest the potential of TNF-a as a surrogate marker for CC screening, most especially in developing countries such as Nigeria where universal HPV vaccination, especially for school-age adolescents, is still lacking. This study is unique in its use of clearly defined case and comparison subgroups, focusing on a particularly vulnerable group of women. However, association studies are devoid of experimental evidence that establishes causation; therefore, further studies are required to determine the association between host factors and TNF-a polymorphism to harness the diagnostic and therapeutic advantage these associations will provide in managing CC.

## Human ethics and consent to participate

The authors thank Dr Aron and other staff of Inqaba Biotechnical Industries (Pty) Ltd. (Pretoria, South Africa) for their help in preparing the DNA samples, and the analyses of TNF-a polymorphism. Finally, the authors appreciate all the participating women without whom this study would have been impossible.

## Consent for publication

Not applicable.

## Availability of data and materials

The datasets used and/or analysed during the current study are available from the corresponding author (KSO) upon reasonable request.

## Conflicts of interest

The authors declare no competing interests in the conduct and publication of the study in this article.

## Author contributions

SOJ-O, ICU and KSO contributed to the study's conception and design. Material preparation, data collection and analysis were performed by SOJ-O, AAA, OA and KSO. The first draft of the manuscript was written by SOJ-O and KSO, and all authors commented on previous versions of the manuscript. All authors read and approved the final manuscript.

## Figures and Tables

**Table 1.** Oligonucleotides probe and sequences.

Name of the Oligo	Sequence of Oligo
rs1800629_F	ACGTTGGATGATCTTTTTCCTGCATCCTG
rs1800629_R	ACGTTGGATGTAGGACCCTGGAGGCTGAAC
rs1800629_E	TCTGTTTCCTTCTAA
rs1799964_F	ACGTTGGATGGGAAGCAAAGGAGAAGCTG
rs1799964_R	ACGTTGGATGCTACATGTGGCCATATCTCC
rs1799964_ E	GAGAAGCTGAGAAGA
rs361525_F	ACGTTGGATGCACACAAATCAGTCAGTGGC
rs361525_R	ACGTTGGATGAAGCATCAAGGATACCCCTC
rs361525_E	GCCCAGAAGACCCCCCTC

Abbreviations: F forward primer, R reverse primer, E extension/mutation primer

**Table 2.** Demographics in the cases and comparison groups (*n* = 75).

Group	*N* (%)	Age (years) (mean ± SD)	*F*	*p*	*P* ^a^	Age (years) at menarche (mean ±SD)	*F*	*p*	*P* ^b^	Age (years) at Coitarche (mean ±SD)	*F*	*p*	*P* ^c^
HIV-/CC+	13 (17.3)	57.62 ± 13.35				15.88 ± 2.48				19.86 ± 2.41			
HIV+/CC+	34 (45.3)	58.00 ± 9.67			0.94	16.00 ± 1.41	2.11	0.12	0.47	23.50 ± 4.95			0.45
HIV+/CC-	17 (22.7)	43.18 ± 9.31	9.66	<0.01		14.00 ± 1.94				20.88 ± 4.66	1.07	0.38	
HIV-/CC-	11 (14.7)	41.82 ± 7.74			0.68	14.09 ± 1.81			0.08	23.13 ± 3.72			0.45

Abbreviations: SD, standard deviation; CI, Confidence interval; HIV negative with cervical cancer, HIV-/CC+; HIV positive with cervical cancer, HIV+/CC+; HIV positive without cervical cancer, HIV+/CC-; HIV negative without cervical cancer, HIV-/CC-

*p*: *p* values for participant ages in cases (HIV-/CC+ and HIV+/CC+) versus comparators (HIV+/CC- and HIV-/CC-)

*p*^a^: *p* values for participant ages in cases (HIV-/CC+ versus HIV+/CC+ subgroups) and comparators (HIV+/CC- versus HIV-/CC- subgroups)

*p*^b^: *p* values for age at menarche in cases (HIV-/CC+ versus HIV+/CC+ subgroups) and comparators (HIV+/CC- versus HIV-CC-subgroups)

*p*^c^: *p* values for age at coitarche in cases (HIV-/CC+ versus HIV+/CC+ subgroups) and comparators (HIV+/CC- versus HIV-/CC-subgroups)

*p* used ANOVA, *p*^abc^ used *T*-test

**Table 3.** Genotypes and allelic distribution of the studied TNFα SNPs.

SNP	Genotype/ allele	HIV-/CC+	HIV+/CC+	HIV+/CC-	HIV-/CC-	*p*-value^a^,	*p*-value^b^	*p*-value^c^
		N (%)	N (%)	N (%)	N (%)	OR (95% CI)	OR (95% CI)	OR (95% CI)
TNF α-308G/A (rs1800629)	GG	10 (76.9)	28 (82.4)	15 (88.2)	9 (81.8)			
	GA	3 (23.1)	5 (14.7)	2 (11.8)	2 (18.2)	0.63	0.73	0.82
	AA	0	1 (2.9)	0	0			
Allele frequency	G	0.88	0.9	0.94	0.91	0.06 0.44 (0.06–3.16)	0.04 1.33 (0.23–7.75)	0.03 0.81 (0.13–4.88)
	A	0.12	0.1	0.06	0.09			
	HWE Test	0.22	1.41	0.07	0.11			
TNF α-238G/A (rs361525)	GG	12 (92.3)	34 (100.0)	16 (94.1)	11 (100.0)			
	GA	1 (7.7)	0	1 (5.9)	0			
	AA	0	0	0	0	1.00	0.33	1.00
Allele frequency	G	0.96	1	0.97	1	0.01 0.75 (0.04–13.2)	Reference	Reference
	A	0.04	0	0.03	0			
	HWE Test	0.02	Reference	0.02	Reference			
TNF α-1031T/C (rs1799964)	TT	10 (76.9)	33 (97.1)	14 (82.4)	9 (81.8)			
	CT	3 (23.1)	1 (2.9)	3 (17.6)	2 (18.2)	1.00	0.10	0.14
	CC	0	0	0	0			
Allele frequency	T	0.88	0.99	0.91	0.91	0.03 1.4 (0.23–8.42)	0.08 0.14 (0.01–1.48)	0.03 1.37 (0.01–1.68)
	C	0.12	0.01	0.09	0.09			
	HWE Test	0.22	0.007	0.16	0.11			

Abbreviations: OR, Odds ratio; CI, Confidence interval; HWE, Hardy-Weinberg equilibrium

HIV negative with cervical cancer, HIV-/CC+; HIV positive with cervical cancer, HIV+/CC+, HIV, positive without cervical cancer, HIV+/CC-; HIV negative without cervical cancer, HIV-/CC-

*p*^a^: *p* values for individual genotype distribution in HIV-/CC+ group versus HIV+/CC- subgroup; *p*^b^: *p* values for individual genotype distribution in HIV+/CC+ versus HIV+/CC- comparison subgroup; *p*^c^: *p* values for individual genotype distribution in HIV-CC+ group versus HIV-/CC- comparison subgroup

*p* used chi-square/fisher exact
